# Hip displacement in children with cerebral palsy: surveillance to surgery – a current concepts review

**DOI:** 10.1051/sicotj/2024023

**Published:** 2024-08-22

**Authors:** Jason J. Howard, H. Kerr Graham, Ashok Johari, Unni Narayanan, Lisa Bennett, Ana Presedo, Benjamin J. Shore, Tatiana Guerschman, Alaric Aroojis

**Affiliations:** 1 Department of Orthopedic Surgery, Nemours Children’s Hospital 1600 Rockland Road Wilmington Delaware 19803 USA; 2 Department of Orthopaedic Surgery, Royal Children’s Hospital 50, Flemington Road Parkville Victoria 3052 Australia; 3 Department of Paediatric Orthopaedics, Children’s Orthopaedic Centre 298, Lady Jamshedji Road Mahim West, Mumbai 400016 Maharashtra India; 4 Division of Orthopaedic Surgery, The Hospital for Sick Children & University of Toronto 555 University Avenue Toronto M5P 3E1 Ontario Canada; 5 Orthos Orthopaedic Solutions Inc. 452 E Columbia St. New Westminster British Columbia V3L 3X5 Canada; 6 Department of Pediatric Orthopedics, Robert Debré University Hospital 48 Blvd Sérurier Paris 75019 France; 7 Department of Orthopaedic Surgery, Boston Children’s Hospital, Harvard Medical School 300, Longwood Avenue Boston MA 02115 MA USA; 8 Department of Paediatric Orthopaedics, Sabara Children’s Hospital Ave. Angélica, 1987 Consolação São Paulo SP 01227-200 Brazil; 9 Department of Paediatric Orthopaedics, Bai Jerbai Wadia Hospital for Children Acharya Donde Marg Parel Mumbai 400012 Maharashtra India

**Keywords:** Cerebral palsy, Hip displacement, Surveillance

## Abstract

This review brings together a multidisciplinary, multinational team of experts to discuss the current state of knowledge in the detection and treatment of hip displacement in cerebral palsy (CP), a global public health problem with a high disease burden. Though common themes are pervasive, different views are also represented, reflecting the confluence of traditional thinking regarding the aetiology and treatment of hip displacement in CP with emerging research that challenges these tried-and-true principles. The development of hip displacement is most closely related to gross motor function, with radiographic surveillance programs based on the Gross Motor Function Classification System (GMFCS), the goal being early detection and timely treatment. These treatments may include non-operative methods such as abduction bracing and Botulinum Neurotoxin A (BoNT-A), but outcomes research in this area has been variable in quality. This has contributed to conflicting opinions and limited consensus. Soft tissue lengthening of the hip adductors and flexors has traditionally been employed for younger patients, but population-based studies have shown decreased survivorship for this treatment when performed in isolation. Concerns with the identification of hip displacement in very young children are raised, noting that early reconstructive surgery has a high recurrence rate. This has prompted consideration of viable minimally invasive alternatives that may have better success rates in very young children with CP, or may at least delay the need for osteotomies. Recent reports have implicated the role of abnormal proximal femoral growth and secondary acetabular dysplasia as a primary cause of hip displacement, related to ambulatory status and abductor function. As such, guided growth of the proximal femur has emerged as a possible treatment that addresses this purported aetiology, with promising early results.

## Introduction

On September 23, 2023, the SICOT Orthopaedic Rehabilitation Committee and SICOT PIONEER organized a webinar on “Hip Displacement in Children with Cerebral Palsy: Surveillance to Surgery” [1], bringing together a multidisciplinary, multinational team of experts to discuss the current state of knowledge in the detection and treatment of hip problems in cerebral palsy (CP). On account of the very favourable response to the webinar, it was decided to convert the proceedings of the webinar into a current concepts review in order to capture the global perspectives of the experts involved. Though common themes are pervasive, different views are also represented, reflecting the confluence of traditional thinking regarding the aetiology and treatment of hip displacement in CP, with emerging research that challenges these tried-and-true principles; most notably, the role of adductor spasticity.

The authors hope that this collection of perspectives, representing views from India, Australia, Canada, France, and the United States, will motivate others to further investigate the topics within, adding to our understanding of the pathophysiology of hip displacement and tailoring our treatments accordingly.

## Hip displacement in children with cerebral palsy – why is it a problem?

Hip displacement is a common problem in CP, its aetiology is characterized by motor weakness, spasticity and muscle imbalance. Because of this, it is easy for hip displacement to develop insidiously, due to progressive deformities in the proximal femur and acetabulum.

### Pathomechanics of hip displacement, clinical correlation and risk factors

Baker et al. [2] stated that every patient with CP has an abnormal hip until proven otherwise and the reasons are not difficult to find. The imbalance of the forces between the spastic hip adductors and flexors against the weak hip abductors and extensors has a role in the development of hip displacement, whereby the femoral head migrates outside the acetabulum. As discussed in later sections, there is increasing interest in the contribution of abnormal proximal femoral growth as a primary aetiology, related to abnormal weight-bearing and abductor weakness. The consequent migration stretches the joint capsule and the connecting ligaments of the femoral head. The degree of displacement is initially small but may progress to dislocation. The speed of femoral head migration can be influenced by a number of factors including the severity of motor involvement, muscle imbalance, and postural factors.

**Figure 1:**
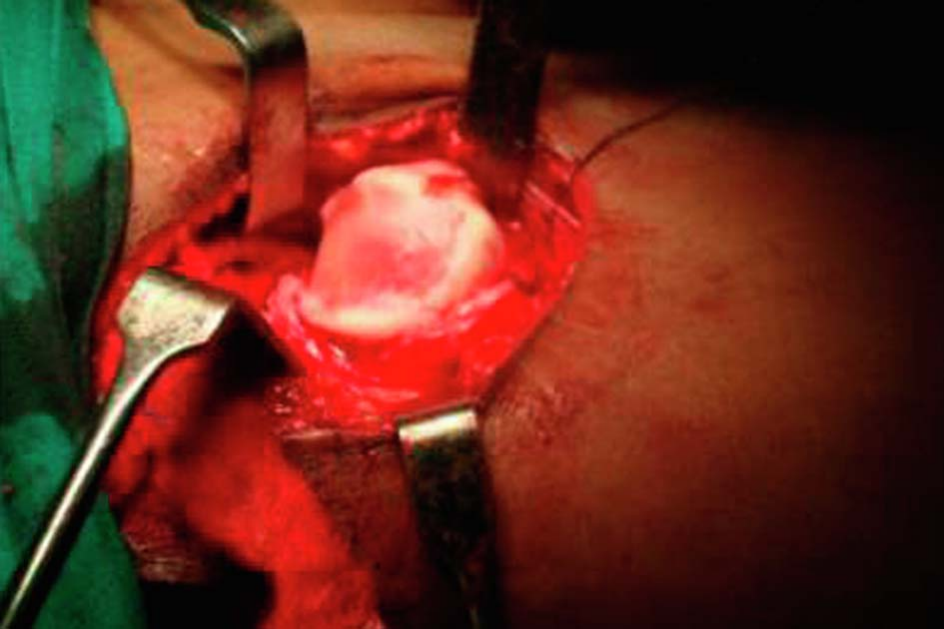
Clinical photo showing end-stage hip disease with gross cartilage loss.

The severity of motor involvement can be stratified based on the Gross Motor Functional Classification System (GMFCS), with the non-ambulant (GMFCS levels IV and V) groups being at the highest risk [3]. The limitation of abduction is a consequence of the adductor tightness. Hip flexor and adductor tightness contribute to displacement and deformation of the femoral head. Femoral head deformation is associated with a loss of articular cartilage and pain which in turn increases the spasticity in a vicious cycle. Activities of daily living, hygiene, and seating become difficult and a patient who could previously ambulate with support may become bedridden. This situation is common for patients with end-stage hip disease, where severe deformities and arthritis may preclude reconstructive osteotomies, necessitating joint resection (i.e., salvage) procedures for pain relief (Figure 1). Hip displacement in CP begins insidiously and is progressive. It rarely improves without treatment. Lack of awareness and knowledge about hip displacement allows it to progress, with detection occurring at a late stage. Hence, it is important to create awareness of this public health issue to the concerned people, their families and involved professionals, allowing for early detection.

**Figure 2:**
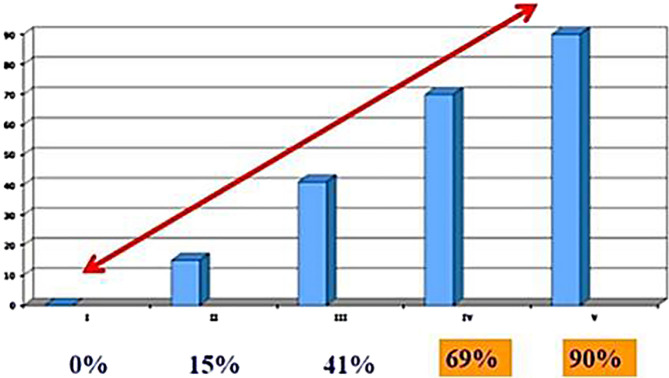
Graph showing direct correlation between hip displacement and GMFCS (adapted from Soo et al. [3]).

Hip displacement has a direct correlation with GMFCS level as has been shown in the study by Soo et al. in 2006 [3] (Figure 2), and subsequently confirmed by many authors. The incidence of hip displacement may be as high as 90% at GMFCS level V. Other potential risk factors for hip displacement include diminishing abduction, pelvic obliquity, progressive scoliosis and limb length discrepancy. Limited hip abduction should arouse suspicion of increasing hip displacement, but this is not ubiquitous. The risk of developing hip displacement is similar for children with hypotonia (without adductor contractures) versus hypertonic motor types (with spastic adductor contractures) [3]. However, this is an important sign when positive.

### Radiology: the migration percentage

The most commonly used index for hip displacement is the migration percentage (MP), as described by Reimers [4]. This is based on the proportion of the femoral head uncovered by the acetabulum. An MP of over 30% is abnormal and deserves close observation. After this threshold has been reached, the hip is considered “at risk” of further progression, necessitating intervention if the migration continues. As the migration percentage increases, unfavourable sequelae such as femoral head deformity may worsen the prognosis of the displaced hip [5].

### Incidence, challenges in detection and role of surveillance

When all that we have mentioned about hip displacement in cerebral palsy is translated to the scenario of a developing country like India, difficult challenges lie ahead [6]. The overall incidence of hip instability in cerebral palsy is quoted to be 35% [7]. Considering the Indian cerebral palsy pool of patients is 2.5 million, there may be 800,000 patients with hip instability; a huge pool to manage for any healthcare system.

Surveillance scrutinizes all aspects of a disease, with a view to prevention, detection and control. Hip surveillance in CP becomes important if we aim to prevent the hip from dislocating. By surveillance, we can detect hip displacement at an earlier stage – clinically silent during this time – to prevent adverse sequelae associated with its natural history. Early treatment can preserve hip health and promote a better health-related quality of life [8]. Hip dislocations can be almost eliminated, as proved in country-based hip surveillance programs in Sweden (CPUP) [9] and Australia [10]. Early detection can translate to the institution of lower morbidity interventions which retain the hip in position, providing higher benefits to the patient.

### So, what should we do?

Our role should be to increase awareness of hip surveillance programs amongst families and healthcare professionals with the goal of detecting hip displacement early and minimising morbidity. With this objective in mind, hip surveillance programs should be set up in all countries.

## Hip surveillance programs: the global imperative

The terms “screening” and “surveillance” are sometimes used interchangeably. Screening typically refers to discrete actions or tests at a single time point (e.g. radiograph of the pelvis), while surveillance is a long-term process, which may include repeated screenings [11, 12]. In the context of hip displacement in cerebral palsy (CP), surveillance involves follow-up of patients at risk of hip displacement for screening, monitoring and triage, and forms the basis for referral or decisions about treatment. Hip surveillance in CP comprises a systematic program of periodic clinical and radiographic assessments of children with CP that aims for early detection of the hip-at-risk, monitoring for progression and early orthopaedic referral to facilitate timely intervention [13]. Hip surveillance is an ongoing process that continues for every child at least until skeletal maturity.

Hip displacement in CP is an important health problem because it is common and if left untreated, may impose a significant impact on the comfort, care, mobility and health-related quality of life of patients with CP [14–16]. The natural history of the condition is reasonably well understood [17, 18]. Hips that displace are at risk for progression from subluxation to dislocation. Progressive displacement of the femoral head leads to abnormal loading of the acetabulum which develops dysplastic changes. The uncovered femoral head is subject to abnormal forces that alter the shape of the femoral head and denude the articular cartilage. The dislocated hip develops osteoarthritis. The bone and cartilage changes are accompanied by contractures of the hip adductors and flexors.

**Figure 3:**
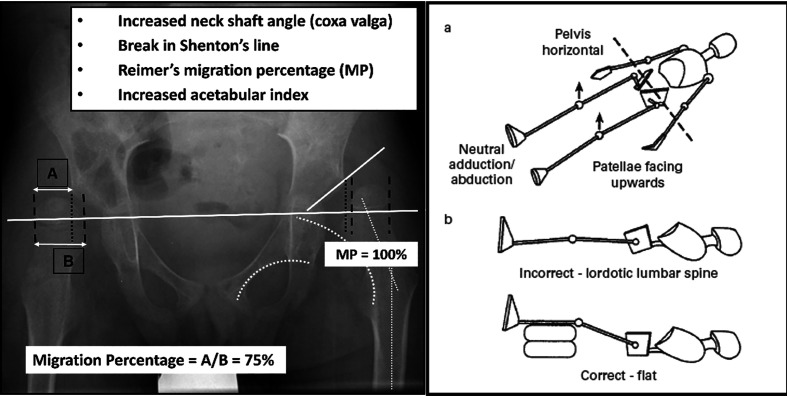
Screening for hip displacement via standardized patient positioning and radiographic examination (adapted from Dobson et al. [19]).

Hip displacement can start early and increase with age, with the highest risk in children with more severe motor involvement [3, 7]. Hip displacement is usually insidious and often asymptomatic until the displacement is more advanced. Clinical examination alone cannot rule out hip displacement. A simple, standardized anteroposterior radiograph of the pelvis is the screening test which is widely available, reliable and accurate and allows the detection and quantification of the magnitude of hip displacement using the Reimer’s Migration Percentage (Figure 3) [4, 20].

There is a general consensus that an MP ≥ 30% or the presence of hip pain meets the threshold for Orthopaedic referral [21, 22]. There is a wide range of treatment options for hip displacement [19]. There is less agreement, however, about the timing and choice of the initial intervention. Early interventions have the advantage of being less invasive, but when done in younger children, are associated with higher recurrence rates necessitating additional surgery [23, 24]. Intervening later will inevitably require more extensive surgery – typically involving femoral and peri-acetabular procedures – which may be more effective in preventing hip dislocations over the longer term [25]. Either approach requires hip surveillance to ensure that intervention can be performed long before the MP approaches 100%. Defining the optimal age and MP to intervene is the goal of the Cerebral Palsy Hip Outcomes Project (CHOP) study, an international multi-centre prospective cohort study that is currently underway [26]. Hip surveillance must be an ongoing process, with the goals of later onset hip displacement, monitoring for progression or recurrence, and helping determine the optimal timing for intervention.

Based on these goals, a number of hip surveillance programs have been established globally [10, 21, 22, 27–30]. These include population-based programs established first in Southern Sweden [expanded to Norway and other parts of Scandinavia and Iceland (CPUP)] [27]. Population-based hip surveillance has also been adopted in Scotland and other parts of the United Kingdom [28], in several Australian states [10] notably Victoria and Tasmania [29], and in the province of British Columbia in Canada [22]. The American Academy of Cerebral Palsy and Developmental Medicine (AACPDM) brought together representatives from each program to develop a unified hip surveillance care pathway that includes evidence- and consensus-based elements [30]. Individual institutions or networks have adopted hip surveillance programs applied to their respective populations.

These hip surveillance programs are similar, sharing common goals of early identification and timely referral to Orthopaedics to facilitate preventative strategies that may reduce the need for surgery, reduce or delay the need for major reconstructive osteotomies, and eliminate the need for salvage surgery [10, 21, 22, 27–30]. The frequency of radiographic evaluation is based on the age and GMFCS levels which together define the risk of hip displacement for children with CP.

Even in the absence of a formal system supported by a health system or institution, individual clinicians who care for children with CP can become familiar with the hip surveillance guidelines and may choose to adopt any one of these existing surveillance protocols and adapt them for their context, and forge relationships with their Orthopaedic colleagues that they can refer patients to for timely management. There is compelling evidence of the success of these programs to significantly reduce the incidence of hip dislocations and the need for salvage operations [9, 10, 28–33]. Although prevention of hip dislocation is an important goal, the intervention or strategy that is the most effective, lasting and efficient means of doing so, remains to be defined.

## Role of non-operative treatment in early hip displacement

Regular hip surveillance by clinical examination and serial radiographs are the key to early diagnosis and treatment. Children with CP must be identified early, as hip displacement can occur as young as 2 years old [7]. Physiotherapy can play a key role in managing hip displacement in CP. The clinical examination in hip surveillance is often the responsibility of the physiotherapist. They classify the child’s GMFCS level, determine the presence and severity of spasticity (typically using the Modified Ashworth Scale), assess a hip range of motion (i.e., abduction), identify pain, evaluate for gait abnormalities, and determine motor type and topographic distribution [22]. Physiotherapists identify infants at risk for motor delays, educate families about their child’s disability, and recommend assistive devices to promote muscle length and joint range. Devices such as robotic gait trainers, that support and facilitate leg movement during stepping, may also have a role [34].

In children with CP, sitting may be compromised by a lack of motor control and weakness that adversely affects trunk and pelvis stability. Sitting can also be affected by spasticity, hypotonia, dystonia, ataxia, lack of head control, and progressive fixed musculoskeletal deformities like scoliosis, hip dislocation, and pelvic obliquity. Seating systems aim to stabilize the pelvis and trunk to provide an upright position of the trunk with a balanced head position, and flexed and slightly abducted hips. Symmetrical alignment helps facilitate optimal postures, which may have beneficial effects in preventing contracture and tone regulation. The aim is free function of the upper extremities, reduced spasticity of the trunk and extremities and sitting tolerance. Positioning systems which prioritize standing with abducted hips, and optimal lying and sitting positions over prolonged periods, may be beneficial in reducing hip displacement [35]. The French approach using siège moulé and gouttière customized plaster cast orthoses for sitting and standing showed a progressive reduction of MP in a few patients [36].

Orthotists work closely with the child and their healthcare team to design orthoses tailored to the child’s unique anatomy and specific needs. Follow-up assessments ensure optimal fit and function. Hip abduction orthoses are designed to maintain the hip range of motion and prevent hip displacement from occurring. There is little high-quality research supporting the use of orthoses for the hip in CP [37]. Kim et al. [38] reported a randomized clinical trial of a novel hip brace that was significantly effective at preventing the progression of hip displacement over the short-term. Long-term studies are required to confirm the durability of this approach.

Botulinum Neurotoxin A (BoNT-A) is commonly used to help manage muscle spasticity [39] and may be used in conjunction with therapy or bracing. Benefits include reduced spasticity, decreased pain, improvements in joint range of motion, and functional ability. A study by Graham et al. in 2006 [23] found the use of BoNT-A combined with an abduction brace delayed progression to surgery but did not prevent progressive hip displacement.

There is a need for more high-quality research of non-operative treatment of hip displacement in CP.

## Soft tissue releases and guided growth – are they effective?

### Indications for treatment of hip subluxation in patients with cerebral palsy

Following Reimers’ work [4], Miller and Bagg evaluated the impact of age and migration percentage (MP) on the risk of hip displacement and established a protocol for treatment [40]. Their results validated the use of this protocol [41, 42]. Since the risk of migration was very low for hips with an MP <30%, remaining stable after the end of growth, the original protocol has been updated [43] (Table 1).

**Table 1:** Protocol for the indication for treatment of hip subluxation in patients with CP.

Age	Migration percentage (MP)	Hip abduction*	Indication
Up to age 8 years	30–60%	<30°	STR**
	30–60%	>30°	Observe
	If MP increases 10%/year	30°–45°	STR**
	>60%		Hip reconstruction
Over age 8 years	>40%	<45°	Hip reconstruction

* Abduction with hip and knee extended.

** Soft tissue releases.

Coxa valga, with a lateral tilt of the proximal femoral physis (quantified by the head shaft angle HSA), appeared as a risk factor for hip displacement [44]. During the past years, several authors recommended guided growth of the proximal femur, combined with soft tissue release (STR, including the adductors), to reduce coxa valga and thus MP. They proposed indications for treatment: HSA>155°, MP 30–50%, and remaining growth ≥2 years [45–47]. Based on these indications, we recently started guided growth, in addition to STR, for children who are between 4 and 10 years of age.

### Surgical technique and postoperative management

Through a transverse inguinal incision, we sequentially section the gracilis, the adductors longus and brevis, aiming for 40° of symmetric abduction. The anterior branch of the obturator nerve may be transected in non-ambulatory patients. Through the same incision, an iliopsoas tenotomy can be performed to treat hip flexion contracture.

**Figure 4:**
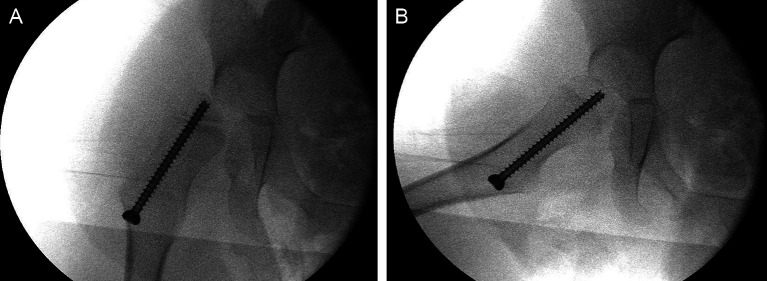
Guided growth combined with STR was indicated for a 4-year-old patient (GMFCS IV) with bilateral MP 50%. Because of the young age, a percutaneous 4.5 mm cannulated screw is centred in the femoral physis in an AP view (A) and centred along the neck in a lateral view (B).

**Figure 5:**
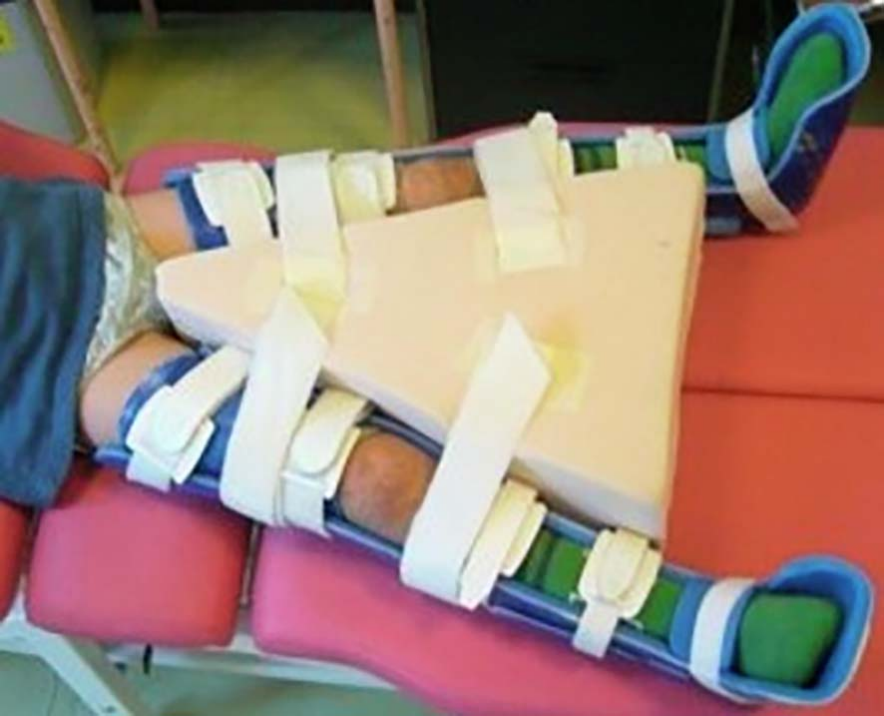
Hip abduction and knee extension are maintained by KAFOs and abduction wedge after STR.

Guided growth of the proximal femur is achieved by inserting a transphyseal percutaneous screw across the inferomedial portion of the proximal femoral physis in an AP fluoroscopic view and centred along the femoral neck in the lateral view (Figure 4). Although the size of the screw depends on the width of the neck, 4.5 mm screws are commonly used for children under 6 years. Also, because of the increased risk of screw grow-off, central placement through the physis has been recommended for young children [48]. Postoperatively, we use splints to maintain knee extension and hip abduction and start immediate physical therapy (Figure 5). Sitting and standing are not restricted. Radiographs are taken at 6 and 12 months after surgery and then scheduled according to hip surveillance protocols [32, 49]. Further treatment recommendations will depend on clinical and radiographic evolution.

### Outcomes

**Figure 6:**
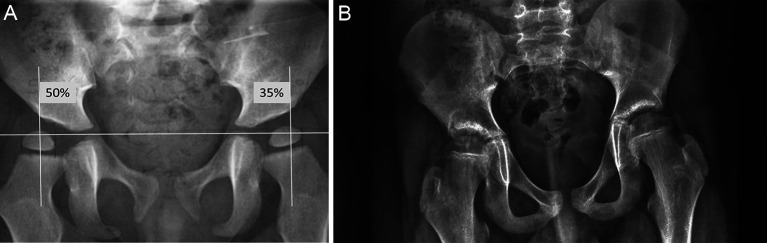
(A) Preoperative radiograph of a 3-year-old patient with spastic quadriplegia (GMFCS III) and hip subluxation with Right MP 50% and Left MP 35%. (B) Ten years after isolated STR showing good outcome.

Despite variable results, we believe that STR can be effective at treating hip subluxation and can decrease the need for extensive surgery in children with CP [24, 41, 42, 50–52]. Adductor surgery is recommended as soon as subluxation is observed (MP >30%) if hip abduction is limited (Figure 6). Factors that correlate with good clinical prognosis have been identified: preoperative acetabular index <34° [52], one-year postoperative MP <30% that remains stable until the end of growth [40, 42, 51]. Overall, with close follow-up and consistent application of standardized treatment protocols, the need for osseous surgery should be reduced to a minimum of 50% of patients with spastic hip disease.

**Figure 7:**
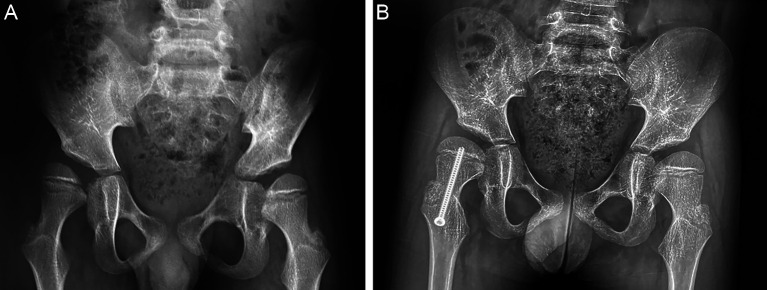
(A) Preoperative radiograph of a 4-year-old patient with spastic quadriplegia and dystonia. Hip abduction was 20° on the right side and 45° on the left side. (B) One year after STR and growth control on the right hip there was improvement of the morphology of the femoral head and acetabulum. Hip abduction was symmetric at 45°.

The addition of growth control to STR has been shown to influence the correction of coxa valga (Figure 7) and diminish the risk of recurrence during the two postoperative years [45–47]. Although no major complications have been reported, it remains unclear to what extent guided growth moderates the influence of age, growth potential and function on proximal femoral development.

## Reconstructive & salvage surgeries for hip displacement

The primary goal of treating hip displacement in children with CP is to maintain flexible, well-located and painless hips with a symmetrical range of motion. Unlike developmental dysplasia of the hip (DDH), children with CP typically are born with normal anatomic hips but due to a combination of delayed motor milestones and asymmetric muscle tone, they develop hip displacement at a later age [53]. Surgical hip reconstruction in non-ambulatory children with cerebral palsy is challenging for the child, the family and the surgeon. The decision when to perform an isolated varus derotational osteotomy (VDRO) of the proximal femur alone, or in conjunction with a pelvic osteotomy, has frequently been discussed in the literature [54, 55]. Once an MP exceeds 50% and there is the presence of hip subluxation/dislocation without degenerative changes to the femoral head, reconstructive hip surgery is recommended [25].

**Figure 8:**
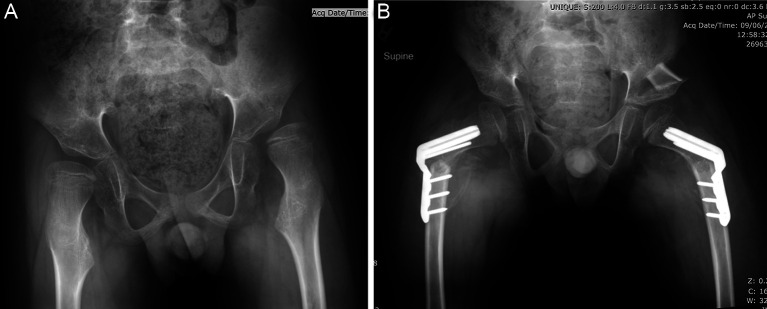
(A) Preoperative radiograph of 9-year-old child with Cerebral Palsy, GMFCS IV with progressive left hip subluxation. (B) Six-week postoperative radiograph demonstrating bilateral femoral osteotomies with cannulated blade plate and a pericapsular osteotomy of Dega on the left side using a wedge of bone from the femur.

Reconstructive options typically include VDRO and pelvic osteotomy (e.g., acetabuloplasty or redirection osteotomy), with or without open hip reduction. Proximal femoral osteotomy allows for simultaneous correction of the increased neck shaft angle and excessive femoral anteversion commonly seen in non-ambulatory children with CP [56, 57]. In some patients, VDRO alone is enough to address hip displacement in children with CP. However, when the displacement is long-standing and associated with significant acetabular dysplasia, consideration of simultaneous pelvic osteotomy is warranted. Intraoperative hip arthrography can help aid in decision-making [58–60]. Once dysplasia has developed, the acetabulum has a limited ability to remodel [61, 62]. Several different acetabuloplasty techniques have been described for the treatment of acetabular dysplasia in children with CP; however, variations of the Dega are widely accepted to generate the most superior results, providing posterior and lateral coverage [54, 59, 63, 64]. The osteotomy should be centred above and directed away from the deficient area to allow for maximal correction at that site (Figure 8). Predictors for failure after unilateral hip reconstruction in non-ambulant children include lack of contralateral soft tissue release, reversal of pelvic obliquity, and an initial migration percentage greater than 25% and younger age (under 8 years) [65].

**Figure 9:**
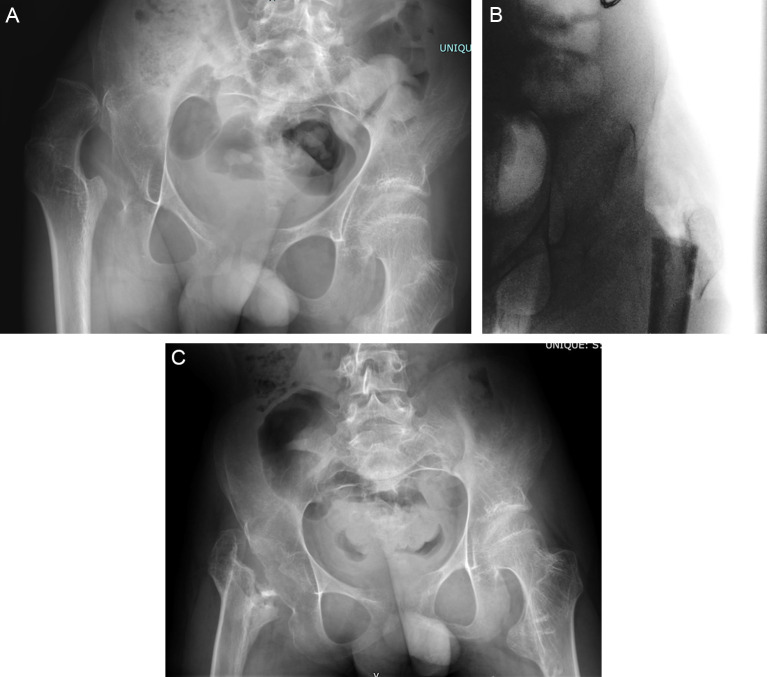
(A) Preoperative radiograph of 18-year-old male, GMFCS III/IV with right hip pain and difficulty with weight bearing and activities of daily living. Note the asphericity of the femoral head and skeletal maturity indicating that remodeling of the femoral head here was not going to be substantive. (B) Intraoperative radiograph demonstrating the relationship between the trochanteric fragment and the femoral shaft. These pieces of bone are transfixed with #2 Fibrewire sutures. (C) Final 1-year radiograph of hip salvage intervention. This young man has returned to weight bearing without pain and his overall function has improved significantly. Note the appropriate station of the proximal femur without significant migration and pelvic support configuration.

Salvage surgery may be indicated for those children and adolescents who present with painful dislocated hips with advanced degenerative changes or when reconstruction has failed, resulting in significant femoral head deformity, articular cartilage loss and pain. Pain and difficulty with perineal hygiene are the two most common indications for surgery in this group of patients. Salvage procedures include proximal femoral resection with soft tissue interposition, valgus osteotomy with femoral head resection, hip arthrodesis, and replacement arthroplasty with mixed results, carrying their own set of risks and potential complications [66–69]. Three recent systematic reviews report heterogeneous studies, with small numbers and short-term follow-ups [70–72]. There are also concerns regarding few studies with appropriate outcomes, such as validated pain scales or quality of life measures. There are few comparative studies and no prospective clinical trials of salvage surgery in the literature. However, recent research using a trans-trochanteric surgical resection has shown promise [73] (Figure 9).

## Long-term outcomes of hip surveillance – how do we temper our approach?

Hip surveillance for children with cerebral palsy (CP) was first practised by Dr Mercer Rang, at the Hospital for Sick Children, Toronto, in the 1970s. Dr Rang established a simple protocol: non-ambulant children with CP and other neuromuscular conditions should have an annual clinical examination of their hips, supplemented by an X-ray. He practised this in his clinics and taught a generation of clinicians about the need for hip surveillance, using posters prominently displayed in the hospital and satellite clinics as well as references in journal articles and books [74, 75]. Rang’s prototypical hip surveillance is similar to protocols used in many countries today [76, 77]. Rang advocated for hip surveillance before the development of the Gross Motor Function Classification System (GMFCS) and at a time when it was felt that a well-timed adductor-psoas release would be sufficient to keep most hips in joint [75, 78]. The development of the GMFCS (also by researchers in Canada), has led to a much better understanding of the risk factors for hip displacement and the natural history [3, 75]. Stratification of surgical outcome studies has shown that GMFCS is a predictor of both the risk of hip displacement and the outcome of both soft tissue and bony surgery [24, 25].

In Table 2, the prerequisites for a successful screening program are summarized from various sources and modified by the authors for hip surveillance in CP. Since the development of the GMFCS, hip surveillance for children with CP fulfils the first four prerequisites but does not meet the crucial 5th prerequisite: “a safe, effective, non-invasive or minimally invasive treatment.” There is no substantive evidence that postural management, seating systems, braces or injections of Botulinum Neurotoxin A (BoNT-A) can prevent hip displacement in children with CP [75]. Indeed, the largest randomised clinical trial of an abduction brace combined with 6-monthly injections of BoNT-A to the hip adductors and hamstrings had no long-term impact in preventing hip displacement or the subsequent need for invasive surgery [23]. Bony reconstructive surgery is effective for the prevention and correction of hip displacement in non-ambulant children with CP but comes at a high cost [75]. There is a high rate of medical and surgical adverse events and occasional mortality, although this is not fully reported [75]. We need new treatment approaches for hip displacement in younger children that are minimally invasive and effective to justify our proactive surveillance programs. Recently, there has been renewed interest in the use of screw epiphysiodesis of the proximal femoral physis to treat hip displacement by reducing coxa valga, with subsequent improvement in hip coverage and reversal of acetabular dysplasia purported. If successful, this guided growth procedure could potentially clear the high bar set by the 5th pre-requisite.

**Table 2:** Pre-requisites for screening programs.

1.	The condition is important with serious consequences to health and wellbeing.
2.	The natural history of the condition has been documented.
3.	There is a pre-symptomatic or early symptomatic stage, when a reliable diagnosis can be made.
4.	There is a diagnostic test that is easy and cost effective to perform and which is reliable, sensitive and specific.
5.	There should be a safe, effective, non-invasive or minimally invasive treatment.

**Figure 10:**
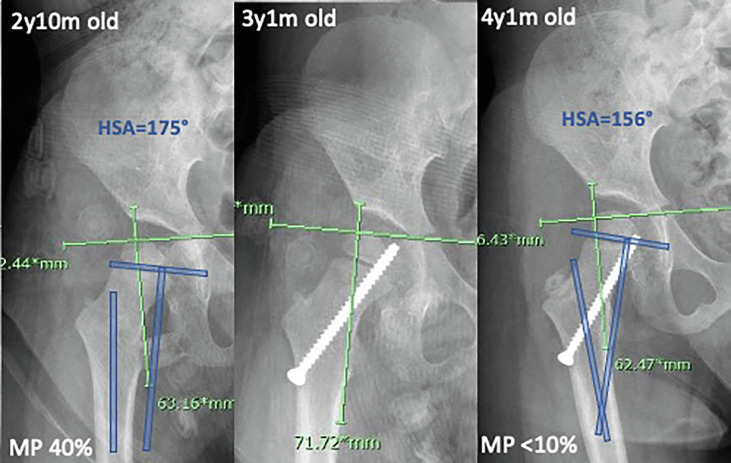
Radiographs showing progressive hip displacement in a child with hypotonic CP (without adductor contractures) treated with guided growth of the proximal femur. Note the reduction in head-shaft angle (HSA, blue lines) and reduction in migration percentage (MP) post-operatively. Change in MP occurred rapidly, likely due to the relatively low pre-operative MP and high growth velocity of the proximal femur at this young age.

Guided growth of the proximal femur has been used to manage Type II growth disturbance of the proximal femur after surgical treatment of DDH [79]. It is now being investigated for both primary and secondary management of hip displacement in children with CP. However, most studies in this area have shown modest gains in the reduction of hip migration, with older children primarily investigated. Emerging data from the use of guided growth in children under age 6 has shown more promising results, with age and severity of pre-operative hip migration being the most important factors in determining the success of this lower-risk, minimally invasive treatment (Figure 10). Comparative, ideally randomized, studies with longer follow-ups are required to fully determine the utility of this technique in CP as well as the predictors of success. Suggested indications for guided growth as a treatment for early hip displacement in CP are provided (Table 3).

**Table 3:** Suggested goals and indications for guided growth treatment of the proximal femur in CP.

Secondary guided growth	Primary guided growth
After hip reconstructive surgery	Before bony surgery
To prevent rebound coxa valga	With/without adductor-psoas release
To reduce or avoid revision surgery	Frail children, GMFCS IV/V
Temporary GG: by inserting a screw	More research before routine use
Permanent GG: “spot weld” physis	Needs RCT and longer-term studies

GG: guided growth; RCT: randomized controlled trial.

## Summary

The development of hip displacement is primarily related to ambulatory status, detected by global radiographic surveillance programs based on the GMFCS, the goal being early identification and treatment. These treatments may include non-operative methods such as bracing and Botulinum Neurotoxin A but research in this area has been either sparse or conflicting. Soft tissue lengthenings of the hip adductors and flexors continue to have a role, but population-based studies have shown decreased survivorship for this treatment approach alone, challenging the role of adductor spasticity as a primary cause of hip displacement.

Concerns with the identification of hip displacement in very young children are valid, noting that early reconstructive surgery has a high recurrence rate. This has prompted consideration of viable minimally invasive alternatives that may have better success rates in very young children with CP, or may at least delay the need for osteotomies. Recent reports have implicated the role of abnormal proximal femoral growth and secondary acetabular dysplasia as a primary cause of hip displacement, related to ambulatory status and abductor function. As such, guided growth of the proximal femur has emerged as a possible treatment which addresses this purported aetiology, with promising early results.

Reconstructive osteotomies performed at the right time can give lasting results and positive effects on quality of life. Salvage procedures involving various methods of proximal femoral resection have less reliable results but can be avoided through the institution of regular radiographic surveillance programs that allow for timely hip management before substantial joint deformity has occurred.

Future studies, ideally population-based, are required to more fully evaluate newer treatments such as guided growth, to preserve hip health at skeletal maturity, allowing for a pain-free, mobile hip in adulthood.

## Data Availability

This article has no associated data generated and/or analyzed.
